# Serum antibodies from Parkinson's disease patients react with neuronal membrane proteins from a mouse dopaminergic cell line and affect its dopamine expression

**DOI:** 10.1186/1742-2094-3-1

**Published:** 2006-01-20

**Authors:** Victor C Huber, Tapan Mondal, Stewart A Factor, Richard F Seegal, David A Lawrence

**Affiliations:** 1 Wadsworth Center, New York State Department of Health, Albany, NY 12201, USA; 2 Parkinson's Disease & Movement Disorders Center, Albany Medical College, Albany, NY 12208, USA

## Abstract

Evidence exists suggesting that the immune system may contribute to the severity of idiopathic Parkinson's disease (IPD). The data presented here demonstrates that antibodies in the sera of patients with IPD have increased binding affinity to dopaminergic (DA) neuronal (MN9D cell line) membrane antigens in comparison to antibodies in sera from healthy controls. In general, the degree of antibody reactivity to these antigens of the mouse MN9D cell line appears to correlate well with the disease severity of the IPD patients contributing sera, based on the total UPDRS scores. Surprisingly, the sera from IPD patients enhanced the DA content of MN9D cells differentiated with n-butyrate; the n-butyrate-differentiated MN9D cells had a greater concentration of DA (DA/mg total protein) than undifferentiated MN9D cells, especially early in culture. Although the IPD sera did not directly harm MN9D cellular viability or DA production, in the presence of the N9 microglial cell line, the amount of DA present in cultures of untreated or n-butyrate-treated MN9D cells was lowered by the IPD sera. The results suggest the involvement of antibodies in the decline of dopamine production and, thus, the potential of immune system participation in IPD.

## Introduction

Idiopathic Parkinson's disease (IPD) is a progressive neurological disorder that affects approximately 1 million people in North America [1,2]. It is characterized clinically by a loss of motor control as evidenced by muscular rigidity, resting tremor, bradykinesia, and gait dysfunction with postural instability [1,2]. Pathological features include, predominantly, the degeneration of dopaminergic (DA) neurons within the substantia nigra (SN) and intracytoplasmic inclusions (Lewy bodies) within surviving neurons [3]. To date, the cause of this disease remains unknown [4]; however, certain gene mutations, e.g., alpha-synuclein, parkin, DJ1, LRKK2, PINK1, and ND5 have been implicated [5]. Expression of any of these mutated genes may enhance the likelihood of IPD by itself or after an environmental insult.

Although potentially only a consequence of IPD pathology, abnormal immune activity has been considered a possible cause of IPD based on post-mortem analysis of IPD patients' brains [6-8] and utilization of mouse models of parkinsonism [9-12]. Specifically, roles for both the innate immune system, as evidenced by increased expression of pro-inflammatory cytokines [10,13-16], and the adaptive immune system, in the form of increased levels of neuron-specific antibodies in the sera of IPD patients [17-24], have been posited.

To date, the strongest evidence for specific immune involvement in the development of IPD was published by Chen et al. when they reported a selective loss of DA neurons within the SN region of rat brains upon administration of immunoglobulin (Ig) G from sera of patients with IPD [25]. Furthermore, in later studies by the same group [26,27], *in vivo *and *in vitro *models demonstrated an important contribution of Fc receptor-bearing cells in the induction of TNF-α, which, in turn, resulted in a reduction of DA neurons as evidenced by decreased tyrosine hydroxylase (TH) activity [26]. However, there have been no reports detailing the specific reactivities of IPD sera with neuronal cell membrane antigens.

In this study, we set out to examine the interaction between antibodies in sera from IPD patients and DA neurons. We determined that serum IgG from IPD patients react with membrane proteins from mouse MN9D neuronal cells to a greater extent than serum IgG from healthy control individuals. Additionally, we found that IPD sera have differential modulatory effects on DA expression by MN9D cells cultured in the presence and absence of N9 microglia. The observed interactions and their possible implications are discussed.

## Methods

### Sera and IPD patients

During a routine office visit, IPD patients were asked if they would consider participation in a research project to evaluate their sera for antibodies to DA neurons *in vitro*. The consent form was approved by the Institutional Review Boards for Human Research of two institutions of the investigators. Most control sera were from the spouses of the IPD patients. Venous bloods were collected in EDTA vacutainers, centrifuged to remove cells, and the sera stored at -20°C until utilized as described. Clinical information regarding the IPD patients is provided (Table 1).

**Table 1:** Clinical Data of IPD patients with High (H), Intermediate (I), or Low (L) Relative Western Analysis Values

Lane	Western Value*	Age	Age at onset	H&Y Stage	UPDRS (total)	UPDRS(motor)
1	L	59	59	1	11	7
2	L	59	58	2	8	6
3	L-I	51	50	2	14	10
4	L	69	65	2	28	18
5	L	67	55	2	30	9
6	H	77	57	2	--	--
7	L-I	80	71	3	33.5	22.5
8	H	79	78	4	64	25
9	L	72	65	2	11	9
10	H	57	51	2	82.5	26.5
11	H	71	64	2	33	12
12	H	69	65	2	20	10
13	L	72	70	1	19	10
14	H	48	43	2	17.5	40.5
15	L	57	53	1	16	7
16	H	72	54	4	84	49
17	H	41	36	3	56	34

* Relative western values are based on the summation of each band intensity above the background level); H, high; I, intermediate; L, low values (see Fig. 2). H&Y is the Hoehn and Yahr scale of Parkinson's disease (Hoehn and Yahr, 1967). UPDRS is the unified Parkinson's disease rating scale.

### Cell lines

The MN9D cell line (provided by Dr. Alfred Heller, Department of Department of Pharmacological and Physiological Sciences, University of Chicago) was derived from rostral mesencephalic tegmentum (RMT) of the 14-day-old embryonic mouse employing somatic cell fusion techniques [28]. This clonal hybrid cell line expresses a high amount of DA, which is efficiently depleted by N-methyl-4-phenylpyridinium ion (MPP+), the active metabolite of the neurotoxin N-methyl-4-phenyl-1,2,3,6-tetrahydropyridine (MPTP). The N9 microglial cell line (provided by Dr. P. Ricciardi-Castagnoli, Department of Biotechnology and Bioscience, University of Milano-Bicocca) was derived by retroviral immortalization of day 13 embryonic mouse brain cultures; they are similar to primary microglia in that, upon activation, they produce proinflammatory cytokines [29] and nitric oxide [30].

### Cell viability

Cell viability was assessed in the separate culture and co-cultures of the MN9D and N9 cells in the absence and presence of the human sera. Viability was determined by a MTT assay as described [31] or by exclusion of propidium iodide assayed by flow cytometry [32].

### Membrane protein isolation

Membrane proteins from MN9D cells were obtained using lysis buffer containing 1.5% Triton X-114 (Sigma, St. Louis, MO), 1 mM MgCl_2 _(Fisher Scientific Co., Fair Lawn, NJ), 5 μg mL^-1 ^each of RNase (Sigma) and DNase (Invitrogen Corporation, San Diego, CA) in cold phosphate-buffered saline (PBS) as previously described [33]. Briefly, cells were treated with this mixture for 15 min on ice with vortexing, and then centrifuged at 27,000 × *g *for 10 min at 4°C to remove nuclei. The supernatants were collected and placed at 37°C for 4 min and centrifuged at 400 × *g *in a swinging bucket rotor for 10 min at 25°C. The pellet containing membrane proteins were resuspended in 200 μL of 10 mM Tris•HCl, pH 7.5, and protein was quantified using the BCA assay (Pierce, Rockford, IL) using bovine serum albumin (BSA) as a protein standard.

### ELISA

ELISA 96-well plates (Corning, Inc., Corning, NY) were coated with 10 μg mL^-1 ^MN9D membrane protein in PBS. Plates were washed with PBS containing 0.1% (v/v) Tween-20 (PBS-T), blocked with 10% fetal bovine serum (FBS) in PBS, washed again, and then sera was added at a 1:100 dilution in 20% normal goat serum in PBS. Plates were again washed, and alkaline-phosphatase-conjugated goat anti-human IgG (H + L) (Jackson Immunoresearch Laboratories, Inc.) (1:10,000 in 10% FBS-PBS) was added. After washing, 1 mg mL^-1 ^p-nitrophenyl phosphate substrate (Sigma) in buffer (0.1 M glycine (Sigma), 1 mM MgCl_2 _(Fisher), 1 mM ZnCl_2 _(Fisher), pH 10.4) was used to measure reactivity. Plates were read at 405 nm on a CERES UV900C microplate reader (Bio-Tek Instruments, Winooski, VT). Sera from 27 individuals, including 19 IPD patients and 8 controls, have been analyzed. ELISA for IL-1β, TNF-a and IL-6 were run as previously described [34] with DuoSets of capture and detection antibodies purchased from R & D (Minneapolis, MN).

### Western blot analysis

MN9D membrane proteins (100 μg) were resolved by SDS-PAGE electrophoresis on a 12 % polyacrylamide gel (single 67 mm loading well) for 2.5 hr at 100 v. The proteins were transferred to a PVDF (Millipore, Bedford, MA) membrane (30 min at 20 v) and blocked with 5% (v/v) fish gelatin (Sigma) in PBS containing 0.05% Tween20 (PBS-T). The blot was washed with PBS-T, and affixed to a slot-blotting apparatus (Bio-Rad) that allows multiple sera to be screened simultaneously. Sera from 17 IPD patients and 2 controls (1:50 dilution in 5% normal goat serum in PBS-T) were applied in separate slots and incubated overnight at 4°C while rocking. The blot was then washed with PBS-T, and incubated with biotin-conjugated goat anti-human IgG (gamma chain specific) (Tago, Inc., Burlingame, CA) (1:5,000 dilution in 5% normal goat serum in PBS-T). The blot was again washed with PBS-T followed by addition of HRP-conjugated streptavidin (1:20,000 dilution; Pierce, Rockford, IL). After a final wash with PBS-T, Super Signal West Pico (Pierce), a chemiluminescent substrate was added. Reactivity was observed with a Fuji LAS 1000 system (FujiFilm Medical Systems USA, Inc., Stamford, CT), and analyzed using ImageGauge software (FujiFilm Medical Systems USA, Inc.).

### Cell culture conditions

MN9D and N9 cell lines were cultured in Dulbecco's modified Eagle's medium with L-glutamine and 4500 mg L^-1 ^glucose, without sodium bicarbonate (Sigma). Importantly, this medium contains pyridoxal•HCl, which is required for the survival of the MN9D mesencephalic cell line [28]. This medium was supplemented with 10% FBS (Hyclone, Logan, UT), 50 U mL^-1 ^penicillin and 50 μg mL^-1 ^streptomycin (Invitrogen Corporation), 3.7 g L^-1 ^NaHCO_3 _(J.T. Baker Chemical Co., Phillipsburg, NJ), and 50 μM 2-mercaptoethanol (Sigma).

Cell culture conditions were set up and analyzed as previously described (Le et al., 2001), with minor modifications. In 24-well tissue culture plates (Corning Inc.), 2 × 10^4 ^N9 cells were seeded for 24 hr at 37°C under 5% CO_2_. After 24 hr, medium was removed, and MN9D cells (4 × 10^4^) in fresh medium were added in the presence of individual human sera (1%) samples. The N9:MN9D ratio was maintained at 1:2, as previously described (Le et al., 2001). Cells were co-cultured for three days.

Differentiation of MN9D cells was performed as described [28] by culturing 4.6 × 10^4 ^cells per well in 4 mL medium in 6-well tissue culture plates (Corning). Cells were exposed to 1 mM n-butyrate (Sigma) throughout the seven day culture and designated wells were harvested daily, beginning with day 2. Medium was replaced every 48 hr with fresh medium containing 1 mM n-butyrate.

Co-cultures of differentiated MN9D cells were established by culturing 9.2 × 10^3 ^MN9D cells in 24-well tissue culture plates (Corning Inc.) in the presence of 1 mM n-butyrate (Sigma) and 1% human sera. After 48 hr, the medium was removed, and fresh medium supplemented with 1 mM n-butyrate and 1% human sera was added. Twenty-four hr later, medium was again removed, and cells were washed twice with 1 mL PBS. Indicated wells received 4.6 × 10^3 ^N9 microglia and 1% human sera in n-butyrate-free medium. Cells cultured in the absence of N9 microglia received 1% human sera in n-butyrate-free medium. Cells were co-cultured for three days.

### Quantification of DA expression

At the end of the specified culturing period, plates were centrifuged at 200 × g for 10 min at 4°C, medium was removed, and cells were washed with 1 mL PBS. Cells were exposed to 1 mL 0.2 M HClO_4 _and sonicated as described. This mixture was then centrifuged to remove proteins from the samples, and DA expression was analyzed using high performance liquid chromatography with electrochemical sensors, and quantified using Waters Millenia software (Waters, Milford, MA), as described [35]. The protein pellet was resuspended in 50 μL 0.5 M NaOH, and protein was quantified using the BCA method with BSA as a standard.

## Results

**Figure 1:**
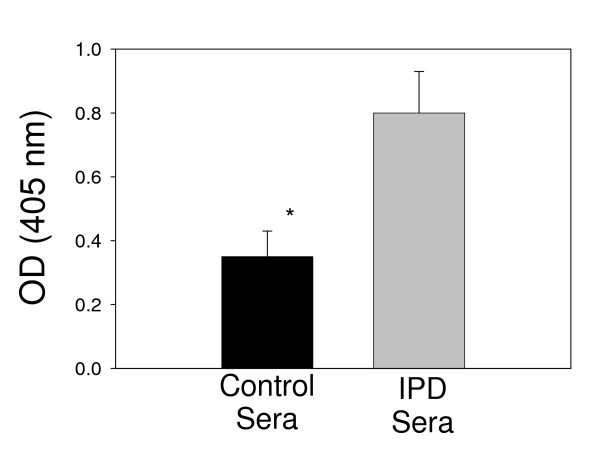
ELISA reactivity of human sera with membrane proteins isolated from MN9D neuronal cells. Black bars represent reactivity of sera from 8 control individuals, and gray bars represent sera from 19 IPD patients. Bars represent the mean and standard error of the mean of five individual experiments. *P < 0.05 compared to control sera.

**Figure 2:**
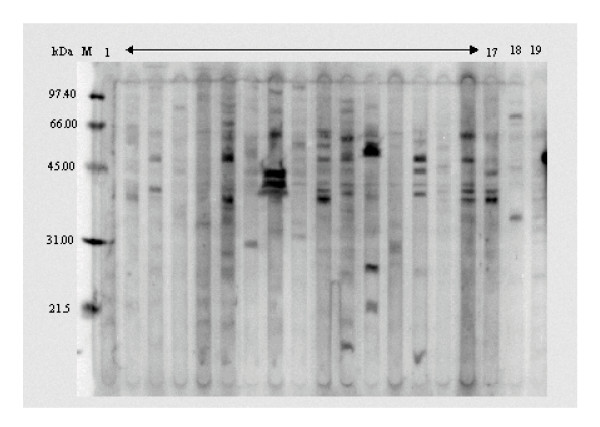
Western blot reactivity of human sera with membrane proteins isolated from MN9D neuronal cells. In this figure, 17 individual IPD sera (lane 1–17) and 2 control sera (lane 18 & 19) were used to probe the PVDF membrane. Numbers designated to the left of the blot reveal the migration of molecular weight standards in kDa. Data are representative of two individual experiments.

When compared with sera from healthy controls, IgG in the sera of IPD patients had significantly increased (*P *< 0.05) binding to ELISA wells coated with MN9D neuronal membrane proteins (Fig. 1). Western blot analysis also demonstrated greater IgG reactivity to MN9D cell membrane proteins (Fig. 2); only two sera from healthy individuals, which had the greatest activity for the control sera, are shown. The Western analysis of the IPD sera revealed antibody reactivity to a number of proteins present in the MN9D neuronal membrane isolates; proteins of 30 to 65 kDa molecular weights were especially predominant. While this reactivity revealed no consistent differences between IPD and control sera and no major common protein band amongst the IPD sera, in general, there was a noticeable increase in the intensity of bands with sera from IPD patients with greater unified Parkinson's disease rating scale (UPDRS) scores (Table 1). The UPDRS is a combined score from the physician's evaluation of motor activity including temors, rigidity, posture, gait, and bradykinesia. For example, the sera from patients 7, 8, 10, 16 and 17 had the most severe IPD (UPDRS-total, 33.5–82.5; UPDRS-motor, 22.5–49), whereas sera from the least severe (UPDRS-total, 8–16; UPDRS-motor, 6–10) IPD patients (1, 2, 9, 15) generally had binding as low as most normal sera.

**Figure 3:**
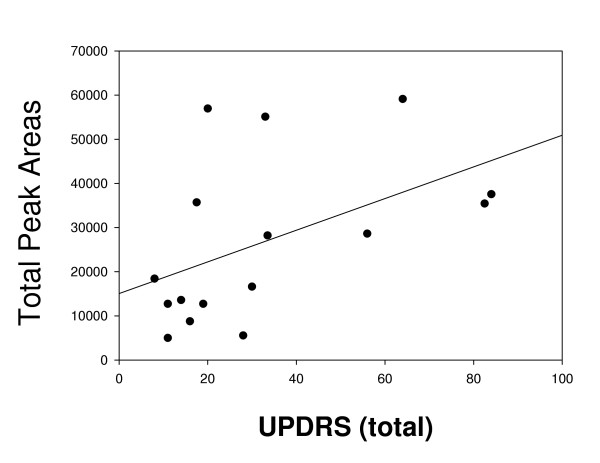
Correlation analysis of clinical score (UPDRS – total) with the sum of the peak areas from the Western analysis (Fig. 2). The r^2 ^value was assessed by linear regression analysis (SigmaPlot 2000) and the significance (*p*) was calculated by Pearson correlation analysis with SigmaStat (Jandel Corp).

Pearson correlational analysis does suggest that the antibodies in the IPD sera significantly correlate (r^2 ^= 0.21; p = 0.05) with the total UPDRS score (Fig. 3). However, there was no correlation of the antibody binding to MN9D antigens (Fig. 2) with regard to UPDRS motor values or the duration of IPD.

To assess if the observed interactions between IPD sera and neuronal antigens correlated with any adverse effects on neuronal cells, *in vitro *assays were performed. This analysis revealed that, compared to control sera, IPD sera had no significant effect on the levels of DA regardless of whether the quantification was calculated as ng/well or ng/mg protein (approximately 10 ng/well and 160 ng/mg protein).

**Figure 4:**
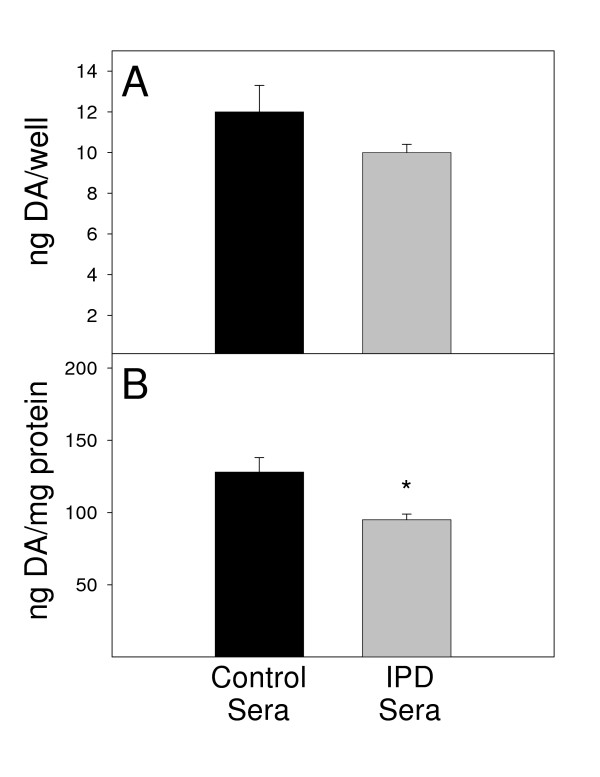
After 72 hr exposure to human sera at 37°C, DA levels were assessed in co-cultures of MN9D neuronal cells (4 × 10^4 ^cells/ml) and N9 microglia (2 × 10^4 ^cells/ml). Black bars represent DA values upon exposure to 8 control sera and gray bars represent values upon exposure to 19 sera from IPD patients. Results are reported as both (A) ng/well and (B) ng/mg protein. Bars represent the mean and standard error of the mean for four individual experiments. *P < 0.05 compared to control sera.

Alternatively, if the N9 microglial cells were co-cultured with the MN9D neuronal cells and sera (Fig. 4), there was a noticeable loss of DA. Although there was not a significant reduction of DA within these co-cultures on a ng/well basis (Fig. 4A), the difference between IPD and control sera became significant (*P *< 0.05) when corrected for protein content (Fig. 4B). Additionally, analysis of the viability of these cells revealed that the observed reductions in DA levels within these co-cultures were not due to the viability of these cells (data not shown). Analysis of the expression of the pro-inflammatory cytokines IL-1β, IL-6, TNF-α, and IFN-γ revealed no difference between IPD and control sera with regard to the expression levels of these cytokines (data not shown). Recent studies have suggested that LPS-activated microglia cause DA neuronal cell death via molecules <350 Daltons, which would rule out cytokines (David Graber, personal communication).

**Figure 5:**
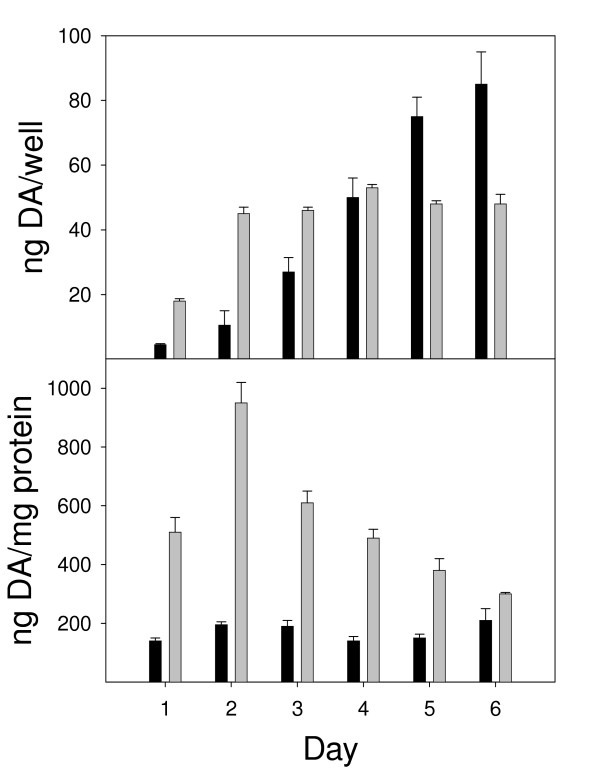
Time course of DA expression by MN9D neuronal cells after exposure to n-butyrate. Black bars represent untreated cells and gray bars represent cells differentiated in the presence of 1 mM n-butyrate. Results are reported as both (A) ng/well and (B) ng/mg protein. Results represent the data from three individual wells per group. *P < 0.05 compared to untreated cells.

It is known that n-butyrate has the ability to induce differentiation of cells *in vitro*, including MN9D cells, as evidenced by an increased number of outgrowths/protections [28] and, as shown here, increased DA levels compared to undifferentiated cells (Fig. 5). DA expression was increased in MN9D cells exposed to 1 mM n-butyrate, compared to undifferentiated MN9D cells on days 2–4 on a ng/well basis (Fig. 5A). However, after Day 4, undifferentiated cells attained the level of DA seen in differentiated cells and, in fact, produced much more DA, comparatively, through Day 7. Differentiated MN9D cell expression of DA plateaued on day 4. Upon correction for protein (Fig 5B), DA values were significantly increased in differentiated cells compared to undifferentiated cells, again peaking at Day 3 and eventually dropping until the level was similar to that seen in undifferentiated cells by Day 7. This data shows that differentiated cells were more effective at producing DA, particularly at Day 3, and for this reason, Day 3 was the day chosen for differentiation of MN9D neuronal cells prior to removal from n-butyrate and exposure to N9 microglia.

**Figure 6:**
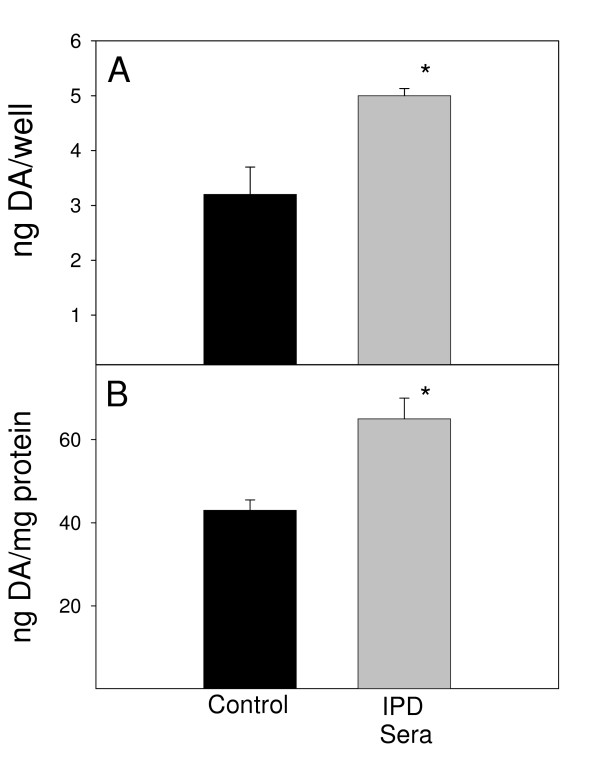
After 72 hr exposure to human sera at 37°C, DA levels were assessed in cultures of n-butyrate-differentiated MN9D neuronal cells (4 × 10^4 ^cells/ml). Black bars represent DA values upon exposure to a pool of 8 control sera; gray bars represent values upon exposure to a pool of 19 sera from IPD patients. Results are reported as both (A) ng/well and (B) ng/mg protein. Bars represent the mean and standard error of the mean for three individual wells. *P < 0.05 compared to control sera.

**Figure 7:**
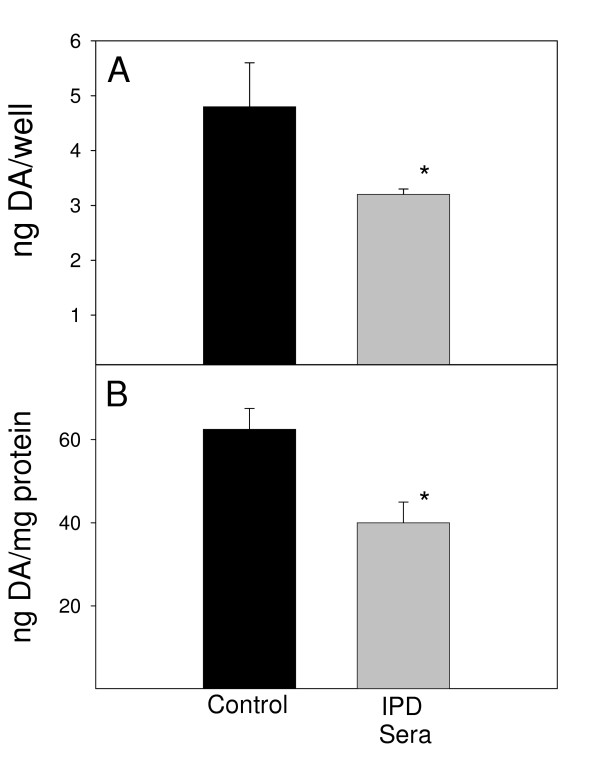
After 72 hr exposure to human sera at 37°C, DA levels were assessed in co-cultures of n-butyrate-differentiated MN9D neuronal cells (4 × 10^4 ^cells/ml) and N9 microglia (2 × 10^4 ^cells/ml). Black bars represent DA values upon exposure to a pool of 8 control sera; gray bars represent values upon exposure to a pool of 19 sera from IPD patients. Results are reported as both (A) ng/well and (B) ng/mg protein. Bars represent the mean and standard error of the mean for three individual wells. *P < 0.05 compared to control sera.

This system of culturing differentiated cells revealed that, when cultured alone, n-butyrate-differentiated MN9D neuronal cells express significantly more DA (*P *< 0.05) in the presence of pooled IPD sera, compared to pooled control sera on both a ng/well and a ng/mg protein basis (Fig. 6). Alternatively, pooled IPD sera decreased DA expression by differentiated MN9D neuronal cells in the presence of N9 microglia (Fig. 7). This difference was not significant on a ng/well basis (Fig. 7A), but was significant (*P *< 0.05) on a ng/mg protein basis (Fig. 7B).

## Discussion

The results reported here reveal the binding interactions between IgG antibodies in the sera of IPD patients and neuronal proteins of a mouse dopaminergic cell line. These associations were examined using ELISA and Western blot techniques, and significant binding of IgG antibodies to DA neuronal antigens was observed. Additionally, there were no adverse effects of the IPD sera in MN9D monocultures, but the IPD sera did significantly decrease the dopamine content of the MN9D cells when N9 microglia were present within the cultures. While there was no evidence that these two activities correlate with any particular antibody specificity, these results suggest that interactions between IPD sera and neuronal cell constituents occur, hinting that antibodies may play a role in IPD. The magnitude of the impact is, as yet, undefined. Furthermore, it is not clear whether these antibodies are involved early in the elicitation of IPD symptoms or arise only after substantial DA neuronal death has occurred.

The reactivity seen between IPD sera and neuronal membrane proteins is striking. The significant differences observed corroborates previously reported data suggesting immunoreactivity between sera and CSF from IPD patients with cellular constituents within the SN of rat brains [17,19-22,24,27]. In fact, the reactivity with the SN was reported to be present in as many as 78% of the CSF samples taken from IPD patients [23]. Surprisingly, aside from a single report describing reactivity of IPD sera with a protein modified by DA oxidation [18], there has been no indication that IPD sera reacts with DA neuronal protein antigens, as is provided in this report. Furthermore, analysis of this reactivity by Western blot revealed a number of proteins that were potentially reactive with both IPD and control sera making it difficult to pinpoint specific proteins that were related to a diseased state. However, the discovery of a number of proteins in the 40–60 kDa range that reacted to a much greater extent with IPD sera than with that of controls further limits the prospective candidate proteins that need to be evaluated.

Previous reports have revealed a specific destructive effect of IPD sera on DA neuronal cells, both *in vivo *[25,27] and *in vitro *[26]. This destructive effect *in vivo *was specific to the SN region of the brain and was only seen when IPD IgG was injected into the SN of rats [25,27]. *In vitro *utilization of a co-culture system to address the effect of these interactions revealed that microglia, activated in the presence of IPD sera have the ability to specifically alter DA neuron function. Induction of TNF-α was previously reported to be the microglial factor involved in the loss of DA [26]; however, we did not observe an increase of TNF-α or any other proinflammatory cytokine in the co-culture supernatants with the IPD sera. It is possible that additional inflammatory microglial products, e.g., nitric oxide and hydrogen peroxide, are responsible for the loss of DA. These small oxidative molecules are likely toxicants and have previously been reported to cause neuronal cell death [36].

Surprisingly, monocultures of the n-butyrate-treated (differentiated) MN9D cells had increased levels of DA when exposed to IPD sera compared to control sera. A possible explanation for this increase is that there is a "damage" signal induced within these neurons upon binding of antibodies to certain MN9D antigens, which are less expressed in the non-treated MN9D cells since they were not affected by the IPD sera in the absence of N9 cells. This positive signal may induce a hyperactivity of the "stressed" differentiated neurons, resulting in increased DA production. Alternatively, antibodies to select surface proteins on the differentiated MN9D cells may directly trigger induction of DA production.

Differences between the non-treated and n-butyrate-treated MN9D cells also were apparent in the presence of the N9 microglia. Significant reductions in DA levels were induced by the IPD sera with co-cultures of non-treated or n-butyrate-treated MN9D cells with N9 cells, when DA was calculated on a ng/mg protein basis. When DA was calculated as DA/culture the non-treated MN9D cocultured with N9 microglia and IPD sera did not have significantly lower levels of DA. This difference is likely due to the fact that the non-treated MN9D cells are proliferating to a greater extent and producing less DA per cell, as suggested by the kinetic analyses shown (Figure 5). Thus, the results with the n-butyrate-treated MN9D cells would more closely represent the *in vivo *situation since normal DA neurons would not be proliferating. The negative microglial effects on DA neurons corroborate the results of Le, *et al*. [26]. It is hypothesized that the antibodies could cross-link the MN9D cells to Fc receptors on the N9 cells leading to release of neurotoxic factors by the N9 microglia.

The positive effect of IPD sera on MN9D monocultures and the negative effect on the co-culture of MN9D and N9 cells with regard to DA production do not address the specific MN9D antigens involved in these processes. However, it is clear that the specificity of the antibodies play a more important role that the amount of antibody, in that when some individual sera were assayed for binding by ELISA and for function (DA levels), there was no correlation. This indicates that the specificity (or possibly isotype) of certain antibodies in the IPD sera and not their concentrations are responsible for altering DA production. This emphasizes the need to further delineate the specific DA neuronal antigens being affected. Analyses are currently underway to isolate and identify the DA neuronal antigens bound by the antibodies, which cause the observed DA changes. In addition to IPD serum antibodies, it is also possible that the IPD sera may contain additional factors affecting DA production, e.g., tetrahydroisoquinolone, β-carbolines. A number of potential toxins could be present in some of the IPD sera [37]. However, this possibility seems unlikely in that the sera were only inhibitory in the presence of the N9 cells.

While attempting to understand the role of the immune system in IPD, we have addressed a number of critical parameters. First, we have shown that there is specific reactivity of sera from IPD patients with multiple membrane proteins expressed by a mouse DA neuronal cell line. Second, we have found neuronal antigen bands of specific reactivity, most notably in the 40–60 kDa range that may lead to further understanding of what neuronal proteins are involved in the antibody-induced alteration of DA production. Finally, through *in vitro *analyses, we show that there is a specific effect of IPD sera on co-cultures of MN9D neuronal cells and N9 microglia, suggesting an interaction between the serum factors and the N9 cells.

## Competing interests

The author(s) declare that they have no competing interests.

## Authors' contributions

VH carried out most of the in vitro analysis and wrote the first draft of the manuscript. TM carried out the Western analyses. SF collected the samples from the IPD patients, provided the information for Table 1, and reviewed the manuscript. RS participated in the design of the study, supervised the HPLC analyses, and reviewed the manuscript. DL conceived of the study, and participated in its design and coordination and helped to draft the manuscript. All authors read and approved the final manuscript.

## References

[B1] Lang A, Lozano A (1998). Parkinson's disease: First of two parts. N Engl J Med.

[B2] Lang A, Lozano A (1998). Parkinson's disease: Second of two parts. N Engl J Med.

[B3] Beal M (2001). Experimental models of Parkinson's disease. Nat Rev Neurosci.

[B4] Olanow C, Tatton W (1999). Etiology and pathogenesis of Parkinson's disease. Annu Rev Neurosci.

[B5] McInerney-Leo A, Hadley D, Gwinn-Hardy K, Hardy J (2005). Genetic testing in Parkinson's disease. Mov Disord.

[B6] McGeer P, Itagaki S, Akiyama H, McGeer E (1988). Rate of cell death in parkinsonism indicates active neuropathological process. Ann Neurol.

[B7] McGeer P, Itagaki S, Boyes B, McGeer E (1988). Reactive microglia are positive for HLA-DR in the substantia nigra of Parkinson's and Alzheimer's disease brains. Neurology.

[B8] Fiszer U (2001). Does Parkinson's disease have an immunological basis? The evidence and its therapeutic implications. BioDrugs.

[B9] Liberatore G, Jackson-Lewis V, Vukosavic S, Mandir A, Vila M, McAuliffe W, Dawson V, Dawson T, Przedborski S (1999). Inducible nitric oxide synthase stimulates opaminergic neurodegeneration in the MPTP model of Parkinson disease. Nat Med.

[B10] Kurkowska-Jastrzebska I, Wronska A, Kohutnicka M, Czlonkowski A, Czlonkowska A (1999). The inflammatory reaction following 1-methyl-4-phenyl-1,2,3, 6-tetrahydropyridine intoxication in mouse. Exp Neurol.

[B11] Teismann P, Tieu K, Choi D, Wu D, Naini A, Hunot S, Vila M, Jackson-Lewis V, Przedborski S (2003). Cyclooxygenase-2 is instrumental in Parkinson's disease neurodegeneration. Proc Natl Acad Sci USA.

[B12] Kohutnicka M, Lewandowska E, Kurkowska-Jastrzebska I, Czlonkowski A, Czlonkowska A (1998). Microglial and astrocytic involvement in a murine model of Parkinson's disease induced by 1-methyl-4-phenyl-1,2,3,6-tetrahydropyridine (MPTP). Immunopharmacology.

[B13] Nagatsu T, Mogi M, Ichinose H, Togari A (2000). Changes in cytokines and neurotrophins in Parkinson's disease. J Neural Transm Suppl.

[B14] Czlonkowska A, Kohutnicka M, Kurkowska-Jastrzebska I, Czlonkowski A (1996). Microglial reaction in MPTP (1-methyl-4-phenyl-1,2,3,6-tetrahydropyridine) induced Parkinson's disease mice model. Neurodegeneration.

[B15] Czlonkowska A, Kurkowska-Jastrzebska I, Czlonkowski A, Peter D, Stefano G (2002). Immune rocesses in the pathogenesis of Parkinson's disease – a potential role for microglia and nitric oxide. Med Sci Monit.

[B16] Nagatsu T, Mogi M, Ichinose H, Togari A (2000). Cytokines in Parkinson's disease. J Neural Transm Suppl.

[B17] Le W, Rowe D, Jankovic J, Xie W, Appel S (1999). Effects of cerebrospinal fluid from patients with Parkinson disease on dopaminergic cells. Arch Neurol.

[B18] Rowe D, Le W, Smith R, Appel S (1998). Antibodies from patients with Parkinson's disease react with protein modified by dopamine oxidation. J Neurosci Res.

[B19] McRae-Degueurce A, Rosengren L, Haglid K, Booj S, Gottfries C, Granerus A, Dahlstrom A (1988). Immunocytochemical investigations on the presence of neuron-specific antibodies in the CSF of Parkinson's disease cases. Neurochem Res.

[B20] McRae D, Gottfries C, Karlsson I, Svennerholm L, Dahlstrom A (1986). Antibodies in the CSF of a Parkinson patient recognizes neurons in rat mesencephalic regions. Acta Physiol Scand.

[B21] Loeffler D, Brickman C, Kapatos G, Peter J, LeWitt P (1992). Anti-Neuronal Antibodies and Other Markers of Immune System Activation in Parkinson's Disease Cerebrospinal Fluid. Neurodegeneration.

[B22] Dahlstrom A, Wigander A, Lundmark K, Gottfries C, Carvey P, McRae A (1990). Investigations on auto-antibodies in Alzheimer's and Parkinson's diseases, using defined neuronal cultures. J Neural Transm Suppl.

[B23] Carvey P, McRae A, Lint T, Ptak L, Lo E, Goetz C, Klawans H (1991). The potential use of a dopamine neuron antibody and a striatal-derived neurotrophic factor as diagnostic markers in Parkinson's disease. Neurology.

[B24] Pouplard A, Emile J (1984). Autoimmunity in Parkinson's disease. Adv Neurol.

[B25] Chen S, Le W, Xie W, Alexianu M, Engelhardt J, Siklos L, Appel S (1998). Experimental destruction of substantia nigra initiated by Parkinson disease immunoglobulins. Arch Neurol.

[B26] Le W, Rowe D, Xie W, Ortiz I, He Y, Appel S (2001). Microglial activation and dopaminergic cell injury: an in vitro model relevant to Parkinson's disease. J Neurosci.

[B27] He Y, Le W, Appel S (2002). Role of Fcgamma receptors in nigral cell injury induced by Parkinson disease immunoglobulin injection into mouse substantia nigra. Exp Neurol.

[B28] Choi H, Won L, Kontur P, Hammond D, Fox A, Wainer B, Hoffmann P, Heller A (1991). Immortalization of embryonic mesencephalic dopaminergic neurons by somatic cell fusion. Brain Res.

[B29] Righi M, Mori L, De Libero G, Sironi M, Biondi A, Mantovani A, Donini S, Ricciardi-Castagnoli P (1989). Monokine production by microglial cell clones. Eur J Immunol.

[B30] Corradin S, Mauel J, Donini S, Quattrocchi E, Ricciardi-Castagnoli P (1993). Inducible nitric oxide synthase activity of cloned murine microglial cells. Glia.

[B31] Matsuki N (2000). Measurement of cellular 3-(4,5-dimethylthiazol-2-yl)-2,5-diphenyltetrazolium bromide (MTT) reduction activity and lactate dehydrogenase release using MTT. Neurosci Res.

[B32] Duncan D, Lawrence D (1991). Residual activation events functional after irradiation of mouse splenic lymphocytes. Radiation Res.

[B33] Narendran A, Hoffman S (1988). Identification of autoantibody reactive integral brain membrane antigens. A two-dimensional analysis. J Immunol Methods.

[B34] Cao L, Lawrence D (2002). Suppression of host resistance to Listeria moncytogenes by acute cold/restraint stress: lack of direct IL-6 involvement. J Neuroimmunol.

[B35] Seegal R, Brosch K, Bush B (1986). High-performance liquid chromatography of biogenic amines and metabolites in brain, cerebrospinal fluid, urine and plasma. J Chromatogr.

[B36] Wang J, Shum A, Ho Y (2003). Oxidative neurotoxicity in rat cerebral cortex neurons: synergistic effects of H2O2 and NO on apoptosis involving activation of p38 mitogen-activated protein kinase and caspase-3. J Neurosci Res.

[B37] Collins M (2002). Alkaloids, alcohol and Parkinson's disease. Parkinsonism Relat Disord.

